# Paretic-Limb-Only Plyometric Training Outperforms Volume-Matched Double-Limb Training for Ameliorating Balance Capability and Gait Symmetry in Adolescents with Unilateral Cerebral Palsy: A Comparative Study

**DOI:** 10.3390/children9101563

**Published:** 2022-10-15

**Authors:** Ragab K. Elnaggar, Reham H. Diab, Asmaa A. Abonour, Saud F. Alsubaie, Saud M. Alrawaili, Mshari Alghadier, Elsayed H. Mohamed, Amira M. Abd-Elmonem

**Affiliations:** 1 Department of Physical Therapy and Health Rehabilitation, College of Applied Medical Sciences, Prince Sattam Bin Abdulaziz University, Al-Kharj 16278, Saudi Arabia; 2 Department of Physical Therapy for Pediatrics, Faculty of Physical Therapy, Cairo University, Giza 12613, Egypt; 3 Department of Basic Sciences, Faculty of Physical Therapy, Cairo University, Giza 12613, Egypt; 4 Physical Therapy Department, College of Applied Medical Sciences, Buraydah Private Colleges, Buraydah 51418, Saudi Arabia

**Keywords:** hemiplegic cerebral palsy, exercise therapy, explosive strength training, dynamic balance, gait performance

## Abstract

Adolescents with unilateral cerebral palsy (U-CP) experience an asymmetrical posture because the less-affected lower limb is preferred for bodyweight support as a strategy of compensating for the paretic side’s muscular weakness. This study was designed to compare the effect of 12 weeks of paretic-limb-only plyometric training (PLPT) and volume-matched double-limb training (DLPT) on balance capability and gait symmetry in adolescents with U-CP. Sixty-nine adolescents with U-CP were randomly assigned to PLPT, DLPT, or a control group (*n* = 23 each). Treatment was delivered twice/week (with at least 48 h recovery intervals) for 12 weeks in succession. The directional (LoS_directional_) and overall (LoS_overall_) limits of stability in addition to the temporal (T-GSI) and spatial (S-GSI) gait symmetry indicis were assessed pre- and post-treatment. The LoS_directional_ improved significantly in the PLPT group compared to either the DLPT or control group (for the forward (*p* = 0.027 and <0.001, respectively), backward (*p* = 0.037 and <0.001, respectively), affected-side (*p* = 0.038 and 0.004, respectively), and less-affected-side (*p* = 0.018 and 0.016, respectively)), and this was also the case for the LoS_overall_ (*p* < 0.001). Additionally, The T-GSI and S-GSI scores decreased significantly in the PLPT group compared to the DLPT (*p* = 0.003 and 0.047, respectively) or control (*p* = 0.003 and 0.036, respectively) group, indicating the development more symmetrical gait patterns. In conclusion, PLPT is likely more effective for enhancing balance capabilities and promoting symmetrical gait patterns than DLPT. Thereupon, it is worthwhile for physical rehabilitation practitioners to include the PLPT paradigm into the intervention plans for adolescents with U-CP.

## 1. Introduction

Cerebral palsy (CP) has become the most prevalent central nervous system condition that results in substantial disability among children/adolescents. Approximately, 2 out of every 1000 live newborns are affected by this condition [1]. Unilateral CP (U-CP), where the motor impairments (i.e., spasticity and paralysis) are typically predominant at one side of the body on account of damage or mal-development of the contralateral cerebral hemisphere, is also regarded as the most frequent subtype of CP, accounting for 33–39% of CP occurrences [2].

Adolescents with U-CP develop an asymmetrical posture because the less-affected lower limb is preferred for bodyweight support as a strategy of compensating for the affected side’s muscular weakness [3,4]. Because of several factors related to increasing energy costs and overuse injuries, this compensating strategy, while effective, may not be efficient [5,6]. Along with the weight-bearing asymmetry, some motor impairments such as increased muscle tone, lack of selectivity, atypical muscle synergies and movements, and contractures of lower limb muscles, are commonly seen in adolescents with U-CP, all of which are thought to contribute to impairment of balance control and development of asymmetrical (temporal and spatial) walking patterns [7,8,9]. In consideration of the foregoing, rehabilitation techniques that promote weight-shifting on the affected lower limb are likely required for adolescents with U-CP to remediate balance incompetence and walking asymmetry, thereby enhancing function [3,10].

Plyometric (Plyo) and explosive strength training are often used interchangeably, are workouts that require muscles to generate the highest force in the shortest amount of time, intending to gain more strength [11]. Such a training model focuses on learning to shift quickly from a muscle extension to contraction and entails executing repeated bodyweight jumping/hopping movements employing the so-called stretch-shortening cycle muscle action [12]. The Plyo activities optimize the neural and musculotendinous systems’ ability to generate maximum force in the fastest way possible, justifying its use, in physical rehabilitation programs, as a link between strength and speed [13]. From this perspective, Plyo training has recently been suggested for the augmentation of motor function in adolescents with U-CP. Prior studies have evidenced that Plyo training can safely and effectively be used for augmenting gross motor function, increasing muscle strength, promoting symmetrical weight-bearing, boosting postural control, and reducing temporo-spatial asymmetrical gait patterns in adolescents with U-CP [3,14,15,16,17,18].

Despite the significance of the findings from prior studies [3,14,15,16,17,18], they have investigated the effectiveness of programs employing either the double-limb Plyo training (DLPT) alone or combined with other exercise approaches, while no studies have specifically been undertaken to compare the effect of the paretic-limb-only Plyo training (PLPT) versus the DLPT in individuals with U-CP. It has been demonstrated that, during explosive motor tasks, force production when muscles of both limbs act simultaneously tends to be lower than the sum of total force produced by each limb acting separately [19]. This is known as the “bilateral deficit”, which is thought to be a result of a reduction in the neuronal drive, a failure to fully engage the muscles of the affected and less-affected limbs when they act at the same time, dependence on the less-affected limb during task performance, and lower active state of muscles in the affected side [3,20]. On that basis, it is intriguing to hypothesize that, in adolescents with U-CP, the PLPT may reduce over-reliance on the less-affected side and allow for larger loads, more strength improvements, and consequently more neural and musculotendinous adaptations in the affected side, as compared with the DLPT, and thereby promoting function and symmetrical performance of the affected and less-affected sides.

Even though PLPT has the potential for conferring greater improvement in motor function, it has not yet been studied in individuals with U-CP. This trial was, therefore, designed to compare the effect of 12-weeks of PLPT and volume-matched DLPT on balance capability and gait symmetry in adolescents with U-CP.

## 2. Materials and Methods

### 2.1. Experimental Design

From January 2019 to August 2020, a three-arm, randomized controlled, clinical trial was undertaken at the Biomechanics Lab and Physical Therapy Center of Prince Sattam in Abdulaziz University (PSAU), Al-Kharj, Saudi Arabia. A single blind protocol was adopted where the outcome assessors were unaware of the intervention allocation for each participant. The study protocol was registered at ClinicalTrial.gov and has been assigned the following identifying number: ID: NCT05302102.

### 2.2. Ethical Considerations

The Institutional Ethical Committee at PSAU approved the study protocol (No: RHPT/0019/0013) on 6 January 2019. Experimental procedures were conducted in accordance with the most recent edition of the Declaration of Helsinki’s ethical guidelines. The willing-to-participate adolescents and their families were informed about the study’s objectives, advantages, and potential hazards. Afterward, before the study began, parents or legal guardians signed a written consent form.

### 2.3. Participants

Sixty-nine adolescents with CP were recruited from the rehabilitation center of the university and tertiary referral hospitals receiving outpatients with neurological deficits in Al-Kharj, KSA. The following were the trial’s inclusion criteria: (1) a confirmed U-CP diagnosis [21]; (2) age from 12 to 18 years; (3) mild spasticity (assessed by the modified Ashworth scale [22] during the outset across the hip extensor/adductor, knee extensor/flexor, and ankle plantar flexor muscles); (4) motor function level I and II based on the Gross Motor Function Classification System [23]; and (5) appropriate intellectual ability—this was verified if the participants were able to accurately perceive, comprehend, and follow instructions during the screening session, attend traditional school classes where they have no trouble with concentrating, communicating, and engaging in traditional learning, and their records indicate normal intellectual function. Adolescents with uncontrolled convulsions, more than 15 mm leg-length discrepancy, irreversible contractures, history of neurotoxins injections or corrective neurological/musculoskeletal surgery in the previous year, uncorrected visual/auditory deficits, or cardiopulmonary comorbidities prohibiting safe engagement in explosive exercises, were excluded.

#### 2.3.1. Sample Size Calculation

To detect clinically relevant changes (i.e., a decisive difference among the study groups), a power analysis was done on a priori basis using the G-Power software (version 3.1.9.7, Neu-Isenburg, Germany). In a 1-way ANOVA study, a sample size of 18 subjects was obtained for each of the three groups whose means are to be compared. The total sample of 54 subjects achieves 92% power to detect a difference in postural control (represented by movement directional control) of at least 5.64% using the Tukey–Kramer (Pairwise) multiple comparison test at a 0.05 significance level. The common standard deviation within a group was assumed to be 2.97%. The sample size was inflated to 69 subjects (23 per group), accounting for a 20% attrition rate.

#### 2.3.2. Assignment Procedure

A two-stage stratified randomization procedure was implemented to assign participants to the three intervention groups (23 participants to each of the PLPT, DLPT, or control groups), thereby allowing to balance the potential prognostic characteristics between groups. Adolescents with U-CP who entered the trial were initially grouped into six strata of varied sizes (according to age and level of motor function). Subsequently, random samples proportional to the size of each stratum were drawn using sealed opaque envelopes, and sub-groups were then combined together to create a sample for each of the intervention groups. The randomization was carried out by a person who was not directly involved in the study and assignment was concealed until assessments and interventions were completed.

### 2.4. Outcome Measures

The same examiner assessed the balance capability and gait symmetry one week before and one week after the intervention while remaining unaware of the intervention that was allocated to each child. Before data collection, all of the participants attended an orientation session to become acquainted with the measuring procedures.

#### 2.4.1. Balance Capability

The dynamic balancing function was assessed utilizing the Biodex balance system (BBS; Biodex Medical System, Shirley, NY, USA). The BBS employs a microprocessor-controlled actuator for controlling the stability of a suspended spherical balance plate. When fully destabilized, the balance plate inclines to 20° at maximum. The system quantifies, in degrees, the individual’s stability by measuring the variance of plate displacement from the level in both the anterior/posterior and medial/lateral directions, at a sampling frequency of 100 Hz. For this study, we used the limit-of-stability (LoS) test to assess the dynamic balancing function [24,25]. This test measures individuals’ capacity to control and move their center-of-gravity (CoG) in various directions across their base-of-support (BoS) without taking steps or losing their balance. The LoS test assesses the time and precision with which the estimated CoG is transferred while moving a cursor to intercept eight consecutive targets arranged around a central target (representing the center of pressure) at 45° intervals and emerge in random order on a display panel. The manufacturer preset the targets to protrude at 50% of the LoS based on the individual’s height.

During the test, participants were instructed to stand barefooted on the balance plate with their feet in a comfortable position their arms by their sides. Thereafter, they were asked to lean their bodies to the maximum extent, while the balance plate was fully destabilized, to reach a target by the cursor, before showing the next target on the display panel. Participants were encouraged to execute the test as accurately and quickly as possible while keeping their bodies straight and using their ankles as primary axes of movement. The test was finished by completing the eight targets. Three attempts were allowed for each child, and mean values were recorded. For data analysis, we used the averaged directional control scores for the forward/backward and affected/less-affected directions. A higher LoS score designates a better balance capability. The following are the algorithms for estimating the directional LoS (LoS_directional_) and overall LoS (LoS_overall_) scores [26]:

$${\mathrm{LoS}}_{\mathrm{directional}}\;\mathrm{score}\;\left(\%\right)=\frac{\mathrm{Straight}-\mathrm{line}\;\mathrm{distance}\;\mathrm{to}\;\mathrm{target}}{\mathrm{Actual}\;\mathrm{distance}\;\mathrm{traveled}}\;\times \;100$$

$${\mathrm{LoS}}_{\mathrm{overall}}\;\mathrm{score}\;(\%)=\sum_{i=1}^{i=4}{\;(\mathrm{LoS}}_{\mathrm{directional}}\;\mathrm{score})\;÷\;4\;(\mathrm{Average}\;\mathrm{of}\;\mathrm{four}\;\mathrm{targets})$$

#### 2.4.2. Gait Symmetry

The GAIT Rite system (GAITRite, CIR System, Clifton, NJ, USA), a portable single layer pressure-sensitive mat (8.3 m long and 0.89 m wide) was used for quantification of temporo-spatial parameters of gait. The pressure sensors are arranged in a network-like pattern with a 12.7 mm separating distance. The mat’s active sensor area measures about 7.32 m long and 0.61 m wide. According to the manufacturer, the system’s sampling frequency is 80 Hz, allowing for an 11-millisecond temporal resolution. The GAITRite^®^ system has been shown to be a valid and reliable instrument for measuring the temporo-spatial parameters of gait [27]. For testing, the mat was set up in a long hallway such that participants were able to walk for two meters before and after the mat, therefore, ensuring a steady-state walking speed across the instrumented portion of the mat. Participants were then instructed to walk at their preferred pace without gait-assistive orthotics. Data from three testing trials were processed through the GAITRite^®^ gold, Version 3.2b software, and the averaged values were employed for the consequent analysis. The single-limb support time (SLSt) and step length (StepL) data were, respectively, used for calculation of the temporal symmetry index (T-GSI) and spatial symmetry index (S-GSI) through the following formulae [8,9,28]. T-GSI = 1 − (SLSt_affected/_SLSt_less-affected_]. S-GSI = 1 − (StepL_affected/_StepL_less-affected_]. Lower T-GSI and S-GSI values are characteristic of enhanced symmetry patterns.

### 2.5. Interventions

#### 2.5.1. Plyometric Training Paradigms

Details of the Plyo training paradigms are depicted in Table 1. Participants in the PLPT and DLPT groups were trained for 45 min, twice/week (with a 48 h recovery-intervals at least), over 12 weeks in succession (totaling 24 sessions), under close surveillance of a certified pediatric physical therapist (i.e., a 1:1 therapist-to-child ratio). All participants were given a pre-practice session to learn how to properly execute exercises. The training focused primarily on the lower extremities and followed the American Academy of Pediatrics’ and the US National Strength and Conditioning Association’s safety performance guidelines [29,30]. The Plyo movements/exercises were performed unilaterally using the affected leg in the PLPT group, and bilaterally through both legs in the DLPT group. On a 4-week basis, the number of exercise sets/repetitions was gradually increased in three blocks, thereby, avoiding adaptation, reducing the risk of overload-induced injury/fatigue, and allowing for a new training stimulus. Exercises in the PLPT or DLPT paradigms were adopted from a series of studies on the effect of Plyo training in adolescents with CP [3,14,15,16]. A standardized 10 min warm-up and cooldown, including static/dynamic stretches, low-intense treadmill walking (at a speed that corresponds to 50–60% of the age-predicted heart rate maximum), and upper limb movements in combination with respiratory exercises were performed before and after the Plyo workout. All Plyo workouts were undertaken on a molded rubbery surface. All exercises were completed while participants were wearing athletic footwear. Participants were urged to optimize their efforts by performing all repetitions in a row with no pauses. A one- or two-minute rest intervals were given between sets.

#### 2.5.2. Standard Physical Therapy

Over the course of 12 weeks, control participants were given the conventional rehabilitation program, which lasted 45 min each session and was repeated two times per week on non-consecutive days. The program was designed to address motor deficits that interfere significantly with daily activities and was overseen by a pediatric physical therapist. Enhancing strength/endurance, improving/maintaining flexibility, boosting postural control, fostering balance, promoting normal gait, optimizing functionality, and minimizing abnormal accommodative patterns have all been prioritized. The program included advanced balance exercises, gait exercises (visually cued on a treadmill and overground), progressive strength training, flexibility, and stretching (functional and active dynamic stretches) exercises [31,32].

### 2.6. Statistical Analyses

The Statistica software, V12 (Statsoft, TIBCO Software, Palo Alto, CA, USA) was used to accomplish all statistical computations. Shapiro–Wilk W test was used to detect deviations from the Gaussian distribution and a logarithmic transformation was used, as necessary. Variables are presented as mean ± StDev unless otherwise specified. The two-way repeated-measures ANOVA test was employed to compute differences between the study groups within the time from the pre- to post-treatment and analyze the group-by-time interactions. Differences between groups were decided by the interaction effects. If there was a significant difference, a pairwise comparison was conducted through Tukey’s post hoc test to determine at which level the effect was different, and a dependent sample *t*-test was used to calculate the changes in each group. The Hedge’s g and partial eta squared (*η*^2^_Partial_) formulae were, respectively, utilized to estimate the size of the significant within-subject and between-group differences. Across all statistical analyses, the null hypothesis was rejected with a *p* value less than 0.05.

## 3. Results

### 3.1. Participants’ Flow and Retention

Ninety-one adolescents were screened for eligibility. Among them, 69 fulfilled the inclusion criteria and were randomly assigned to the study groups. Five participants (~7%; one from the PLPT group, two from the DLPT group, and two from the control group) were lost during the study or missed the follow-up assessments for undisclosed personal issues or scheduling difficulties. Even so, per the intention-to-treat principle, data of all participants (including those who were lost) have been included in the analysis, where missing data were substituted by the pre-treatment observations.

### 3.2. Treatment Compliance, Tolerability and Safety

An equivalent compliance to treatment rate (i.e., the percentage of training sessions that participants actually attended out of the 24 scheduled during a 12-week period) was detected between the study groups (*p* = 0.473). The median (25–75th percentile) of the compliance rate was 95.83% (91.67–95.83%) for the PLPT group, 95.83% (91.67–100%) for the DLPT group, and 91.67% (87.50–95.83%) for the control group. The training was bearable for all of the participants in the either the PLPT group or the DLPT group, and participants in both were all capable of to completing all exercises each session, and none of them reported any adverse effects.

### 3.3. Baseline Characteristics

Characteristics of participating adolescents are shown in Table 2. There were no significant baseline differences (*p* > 0.05) between the study groups as regards all measures of the demographic (age and frequency distribution of gender), anthropometric (height, weight, and body mass index), and clinical characteristics (the affected side, severity of spasticity, level of motor function).

### 3.4. Pre-to-Post Change Differences between Groups

Outcomes of balance capability are presented in Table 3. For the LoS_directional_, there was a significant large 2-way (group × time) interaction effect on LoS_affected_ (*F*_2,66_ = 12.46, *p* < 0.001, *η*^2^_Partial_ = 0.27), LoS_less-affected_ (*F*_2,66_ = 18.77, *p* < 0.001, *η*^2^_Partial_ = 0.36), LoS_forward_ (*F*_2,66_ = 10.89, *p* = 0.0001, *η*^2^_Partial_ = 0.24), and LoS_backward_ (*F*_2,66_ = 10.09, *p* = 0.0002, *η*^2^_Partial_ = 0.23). The post hoc Tukey’s pairwise comparison revealed that changes in the PLPT group were more conducive in comparison with either the DLPT or control group for LoS_affected_ (*p* = 0.038 and 0.004, respectively), LoS_non-affected_ (*p* = 0.018 and 0.016, respectively), LoS_forward_ (*p* = 0.027 and <0.001, respectively), and LoS_backward_ (*p* = 0.037 and <0.001, respectively). For the LoS_overall_, there was also a significant large 2-way interaction effect (*F*_2,66_ = 38.36, *p* < 0.001, *η*^2^_Partial_ = 0.54). The post hoc analysis indicated more favorable changes in the PLPT group as compared with either the DLPT group or control group (*p* < 0.001 for both).

Measures of gait symmetry are shown in Table 4. There was a significant large group-by-time interaction effect on both the S-GSI (*F*_2,66_ = 3.99, *p* = 0.023, *η*^2^_Partial_ = 0.11) and the T-GSI (*F*_2,66_ = 9.14, *p* < 0.001, *η*^2^_Partial_ = 0.21). Per the Tukey’s post hoc analysis, the PLPT group showed lesser S-GSI and T-GSI scores when compared with either the DLPT group (*p* = 0.047 and 0.003, respectively) or control group (*p* = 0.036 and 0.003, respectively), suggesting the development of more symmetrical spatial and temporal patterns.

## 4. Discussion

The purpose of this trial was to compare the effect of the PLPT against the DLPT training on motor function in adolescents with U-CP. In this respect, certain proxies of balance control (LoS_directionl_ in the forward, backward, affected, non-affected side directions, along with the LoS_overall_) and gait symmetry indices (i.e., S-GSI and T-GSI) were assessed in adolescents who either received 12 weeks of Plyo training performed unilaterally using the affected leg in the PLPT group or bilaterally through both legs in the DLPT group. The trial’s most remarkable findings were that the PLPT resulted in larger increases in the LoS in all directions and overall relative to the DLPT, suggesting a better development of the balance capabilities. Another important finding was that the PLPT led to a greater reduction in the disparities in StepL and SLSt between the affected and non-affected sides as opposed to the DLPT, resulting eventually in enhanced patterns of gait symmetry (as clearly proven by the lower S-GSI and T-GSI scores).

Several studies have highlighted the value of Plyo training for children/adolescents with U-CP [3,14,15,16]. Nevertheless, there have been no controlled studies that compared the effects of PLPT versus DLPT training among these patients. This likely underlines the significance of the current findings, which provide clinicians and physical therapists with empirical evidence on a likely effective and novel training paradigm for improving rehabilitation outcomes in adolescents with U-CP. In this trial, the minimum effective dose (12 weeks) and frequency (twice per week) of explosive strength training were adopted according to the NSCA guidelines [30], taking several factors into consideration. First, is the physically demanding nature of Plyo workouts. Compared to standard forms of strengthening exercise, Plyo training can be associated with increased mechanical loading in addition to increased afferent input [12]. Hence, it was particularly essential to consider adequate time for the musculoskeletal system recovery and promoting effective neuromuscular adaptations, while minimizing the risk of injury. Second, the relatively lower physical competencies of the target population (i.e., adolescents with U-CP) compared to those who do not have a motor disability. Third, the research team hypothesized that increasing the dose and frequency of training might cause undue fatigue, thereby, negatively affecting performance and adherence to the training. After all, available evidence on the role of Plyo training in individuals with U-CP demonstrated that 8–12 weeks of training were enough to yield significant changes in several aspects of motor function such as muscle strength, postural control, weight-bearing symmetry, temporal/spatial gait symmetry, response-capacity to balance threats, and functional ability [3,14,15,16,17,18].

Even though the trial’s methodology did not investigate the specific mechanism whereby the PLPT improved balance capability and gait symmetry in individuals with U-CP, several reasonable explanations could be suggested for these distinctive effects. The nature of the PLPT probably played a significant role. Such a training paradigm may have led to further displacement of the body’s center-of-mass in different directions, which presented a wide spectrum of balance challenges and resulted in neuromuscular adaptations that reinforced balance abilities [12,14,33]. Directing training toward the affected leg might have also contributed to enhanced balance by increasing the kinesthetic awareness of that side. The repeated and vigorous stimulation of the mechanical receptors and rapid change in muscles’ length and tension during training could have increased the proprioceptive feedback to the central nervous system [34], which is highly needed for promoting balance competencies [25]. Although not explicitly observed in the current study, performing Plyo training unilaterally through the affected leg could have led to significant improvement in selective motor control (i.e., motor unit recruitment) and consequently muscle strength, which may have become close to the muscle strength in the non-affected side [3,14], and thereby have contributed together to enhanced balance capabilities. It can further be claimed that unilateral training of the affected side and the resultant increases in muscle strength and kinesthetic sensation have promoted the dissemination of the bodyweight on that side and diminished the over-reliance on the non-affected side, which may have resulted in enhanced postural stability [3]. In light of the foregoing discussion, it can ultimately be inferred that these responses (i.e., enhanced strength, kinesthetic sensation, weight acceptance, and balance capability) have probably contributed to a more stable stance and lesser inter-limb discrepancies regarding step length and swing duration, thus allowed the development of symmetrical spatial and temporal gait patterns [14,26].

One of the trial’s main strengths is that it was the inaugural study to demonstrate the efficacy of PLPT in individuals with U-CP. Additionally, the trial’s design (randomized, controlled assessor-blinded trial) with a sufficiently large sample size and reasonably high power (92%) might have increased the probability of making a correct decision about the intervention effects. Further, the key clinical issues of adolescents with U-CP have been covered in the therapeutic focus and response variables (in other words, measured outcomes reflected different function and activity domains, and the training program was geared toward optimization of these measures). In spite of that, a number of limitations should be taken into account when interpreting the current findings. For instance, this trial has been limited to adolescents with U-CP who experience a comparatively better motor capacity than other subtypes of CP, making these findings less generalizable to all individuals with CP. So, this could be a direction for further investigations. Additionally, a specific age range was considered in this trial (i.e., 12–18 years). To establish conclusive evidence, additional studies are, therefore, need to be done to identify the role of PLPT for individuals with U-CP in different age brackets. Further, changes in muscle strength were not assessed in this trial. Whereas muscle weakness is a key factor that considerably impacts balance capacity and gait performance [8,14], further work incorporating this variable is required to fully understand the causative links between muscle strength and balance and gait performance. Furthermore, there were no follow-up measurements beyond the post-treatment occasion in this trial. Thence, more research into the long-term consequences of comparable Plyo training regimens in adolescents with U-CP is warranted to identify how long effects might last.

## 5. Conclusions

The relevance of PLPT for adolescents with U-CP is clearly supported by the current findings. Obtained data showed that PLPT is more effective for enhancing balance capabilities and promoting symmetrical spatial and temporal gait patterns than volume-matched DLPT training. Thereupon, it is worthwhile for physical rehabilitation practitioners to include the PLPT paradigm into the intervention plans for adolescents with U-CP.

## Figures and Tables

**Table 1:** Specifics of the paretic-limb-only/double-limb plyometric training paradigms, performance instructions, and training progression.

**Exercise**	**Exercise Characterization and Directives for Performance**	**1st Block** **(Wk. 1–4)**	**2nd Block** **(Wk. 5–8)**	**3rd Block** **(Wk. 9–12)**
Paretic-limb-only plyometric training paradigm				
Single-leg push-off	− Stand on the floor with the affected foot resting on a 20 cm-high box. − Push off with the affected foot on top of the box, trying to gain as much height as possible by extending through the hip and knee. − Return the non-affected foot to the start position by landing with the affected foot on top of the box.	2 sets—10 reps	3 sets—15 reps	4 sets—15 reps
Lateral push-off	− Stand sideways with the affected foot upon the box. − Push off with the affected foot and jump across the box. − Return the non-affected foot to the start position by landing with the affected foot on top of the box.	2 sets—10 reps	3 sets—15 reps	4 sets—15 reps
Jump split squat	− Stand upright with your feet together and arms by sides. − Jump up and land in a split squat position so that the affected leg is forward with the knee bent and the non-affected leg is backward standing on your toes. − Return to the starting position and repeat, keeping the affected leg forward	1 set—10 reps	2 sets—15 reps	3 sets—15 reps
Single-leg vertical jump	− Stand on the affected leg. − Squat down quickly by bending knee and hip while extending the non-affected leg back and swinging arms back. − Jump upward immediately while swinging the non-affected leg and arms forward and upward. − Lower arms down to sides and land on the affected foot, bending hip, knee, and ankle to absorb impacts.	3 sets—5 reps	4 sets—5 reps	5 sets—10 reps
Single-leg tuck jump	− Stand on the affected leg and raise the non-affected (bent at the knee). − Jump up straight off the ground with the affected leg, bringing the jumping knee higher than the stationary knee. − When the affected leg lands, jump straight back up again with the affected leg.	3 sets—5 reps	4 sets—5 reps	5 sets—10 reps
Double-limb plyometric training paradigm				
Double-leg hop	− Stand with feet together and arms straight out forward. − Bend knees and quickly jump upwards and forward as far as possible. Concentrate on maximizing the distance traveled forward, staying low to the ground. − Repeat jumps immediately upon landing.	2 sets—10 reps	3 sets—15 reps	4 sets—15 reps
Side-to-side jump	− Stand straight with feet hip-width apart and arms by sides. − Bend knees to squat straight down and jump quickly with both feet to the right and then to the left. − Land softly and absorb impacts by squatting slightly into the landing. − Repeat jumping right and left while keeping shoulders and hips facing forward.	2 sets—10 reps	3 sets—15 reps	4 sets—15 reps
Reciprocal stride-jump	− Stand on both legs, with one foot in front, keeping your weight on both feet. − Jump up, with feet (affected/less-affected) interchangeably advanced forward between jumps. − Swing arms, each with the contralateral forward-advanced leg.	1 set—10 reps	2 sets—15 reps	3 sets—15 reps
Double-leg vertical jump	− Stand upright with feet about shoulder-width apart and knees slightly bent. − Drop down the body 10 to 12 inches by flexing knees and explode upward rapidly. Swing arms upward forcefully and reach as high as possible. − Repeat the drill immediately upon landing.	3 sets—5 reps	4 sets—5 reps	5 sets—10 reps
Double-leg tuck jump	− Stand upright with knees slightly bent and feet about shoulder-width apart. − Drop down the body 10 to 12 inches by flexing knees and explode upward rapidly, while swinging arms upward forcefully. − Pull knees immediately up, toward the chest, grab knees with hands, and release. − After landing, repeat the drill immediately.	3 sets—5 reps	4 sets—5 reps	5 sets—10 reps
− Training intensity in each block is expressed as the number of sets/repetitions. − All Plyo workouts were undertaken on a molded rubbery surface. All exercises were completed while participants were wearing athletic footwear. − Participants were urged to optimize their efforts by performing all repetitions in a row with no pauses. − Between training sets, 1- or 2-min rest intervals were permitted.				

**Table 2:** Characteristics (demographic and clinical) of participants in the study groups.

	PLPT Group (*n* = 23)	DLPT Group (*n* = 23)	Control Group (*n* = 23)	*p*- Value
Age, year	14.61 ± 1.70	14.52 ± 1.38	15.26 ± 1.81	0.26 ^‡^
Gender (M/F), *n* (%)	17 (73.9)/6 (26.1)	13 (56.5)/10 (43.5)	15 (65.2)/8 (34.8)	0.52 ^§^
Weight, Kg	50.26 ± 6.87	51.74 ± 6.52	53.87 ± 7.01	0.2 ^‡^
Height, m	1.52 ± 0.11	1.54 ± 0.10	1.57 ± 0.11	0.36 ^‡^
BMI, Kg/m^2^	21.63 ± 1.45	21.82 ± 1.33	21.91 ± 1.29	0.77 ^‡^
Side affected (RT/LT), *n* (%)	5 (21.7)/18 (78.3)	4 (17.4)/20 (82.6)	7 (30.4)/16 (69.6)	0.68 ^§^
MAS level (1/1+), *n* (%)	12 (52.2)/11 (47.8)	14 (60.9)/9 (39.1)	13 (56.5)/10 (43.5)	0.95 ^§^
GMFCS level (I/II), *n* (%)	18 (78.3)/5 (21.7)	11 (47.8)/12 (52.2)	16 (69.6)/7 (30.4)	0.1 ^§^

***Note****:* Numerical data shown as mean ± StDev and categorical data expressed as frequency (%). ***Abbreviations****:* PLPT: paretic-limb-only plyometric training, DLPT: double-limb plyometric training, BMI: body mass index, M/F: male/female *p* values: ^‡^ 1-way ANOVA test, ^§^ Fishers’ exact test.

**Table 3:** Differences in balance capability outcomes among the study groups.

	PLPT Group (*n* = 23)	DLPT Group (*n* = 23)	Control Group (*n* = 23)	Interaction Effect	
				*p*-Value	*η* ^2^_Partial_
**LoS_affected_**					
Pre	47.61 ± 10.86	45.22 ± 9.16	43.96 ± 8.89	<0.001	0.27
Post	58.87 ± 9.29	48.91 ± 8.41	46.35 ± 6.37		
*p*-value	<0.001	0.002	0.06		
Hedges’ g (95% CI)	1.07 (0.64–1.58)	0.41 (0.16–0.67)	––		
LoS_non-affected_					
Pre	55.30 ± 6.59	53.48 ± 6.01	54.17 ± 6.10	<0.001	0.36
Post	64.22 ± 7.29	56.69 ± 4.67	55.83 ± 5.27		
*p*-value	<0.001	.0001	.002		
Hedges’ g (95% CI)	1.23 (0.75–1.80)	0.58 (0.29–0.90)	0.28 (0.11–0.47)		
LoS_forward_.					
Pre	43.39 ± 6.61	42.74 ± 3.92	40.74 ± 4.49	0.0001	0.24
Post	52.10 ± 7.77	45.35 ± 4.30	43.13 ± 4.04		
*p*-value	<0.001	0.0003	<0.001		
Hedges’ g (95% CI)	1.17 (0.61–1.78)	0.61 (0.28–0.97)	0.54 (0.28–0.83)		
LoS_backward_					
Pre	42.52 ± 4.32	41.22 ± 5.96	40.48 ± 3.30	0.0002	0.23
Post	49.57 ± 5.61	44.87 ± 4.15	41.87 ± 3.42		
*p*-value	<0.001	0.001	0.058		
Hedges’ g (95% CI)	1.36 (0.83–1.97)	0.69 (0.27–1.13)	-		
LoS_overall_					
Pre	47.21 ± 4.42	45.66 ± 3.40	44.84 ± 3.44	<0.001	0.54
Post	56.18 ± 3.95	48.96 ± 2.74	46.79 ± 3.10		
*p*-value	<0.001	<0.001	0.0005		
Hedges’ g (95% CI)	2.10 (1.40–2.87)	1.03 (0.64–1.49)	0.57 (0.25–0.93)		

Pre- and post-intervention data are demonstrated as mean ± StDev. * significant at *p* < 0.05, *η*^2^_Partial_: ANOVA’s effect size, Hedges**’** g: *t*-test’s effect size. ***Abbreviations***: PLPT: paretic-limb-only plyometric training, DLPT: double-limb plyometric training, LoS: limit of stability.

**Table 4:** Variations in gait symmetry outcomes between the study groups.

	PLPT Group (*n* = 23)	DLPT Group (*n* = 23)	Control Group (*n* = 23)	Interaction Effect	
				*p*-Value	*η* ^2^_Partial_
StepL_affected_, m					
Pre	0.44 ± 0.06	0.45 ± 0.06	0.44 ± 0.05	<0.001	0.39
Post	0.53 ± 0.07	0.46 ± 0.05	0.45 ± 0.04		
*p*-value	<0.001	0.19	0.002		
Hedges’ g (95% CI)	1.33 (0.76–1.98)	-	0.21 (0.05–0.38)		
StepL_non-affected_, m					
Pre	0.50 ± 0.06	0.49 ± 0.05	0.51 ± 0.04	0.018	0.11
Post	0.56 ± 0.06	0.51 ± 0.04	0.52 ± 0.06		
*p*-value	0.001	0.23	0.49		
Hedges’ g (95% CI)	0.97 (0.42–1.56)	-	-		
SLSt_affected_, sec.					
Pre	0.41 ± 0.05	0.39 ± 0.07	0.38 ± 0.10	0.014	0.12
Post	0.50 ± 0.09	0.44 ± 0.05	0.43 ± 0.07		
*p*-value	<0.001	0.001	<0.001		
Hedges’ g (95% CI)	1.19 (0.72–1.74)	0.79 (0.35–1.28)	0.56 (0.31–0.84)		
SLSt_non-affected_, sec.					
Pre	0.62 ± 0.14	0.61 ± 0.15	0.65 ± 0.16	0.39	-
Post	0.54 ± 0.08	0.56 ± 0.07	0.59 ± 0.11		
*p*-value	<0.001	0.018	0.0002		
Hedges’ g (95% CI)	0.68 (0.36–1.03)	0.41 (0.06–0.78)	0.42 (0.19–0.67)		
S-GSI					
Pre	0.11 ± 0.09	0.12 ± 0.08	0.14 ± 0.11	0.023	0.11
Post	0.04 ± 0.03	0.11 ± 0.05	0.13 ± 0.07		
*p*-value	<0.001	0.64	0.74		
Hedges’ g (95% CI)	1.01 (0.41–1.66)	-	-		
T-GSI					
Pre	0.32 ± 0.11	0.33 ± 0.15	0.39 ± 0.17	<0.001	0.21
Post	0.10 ± 0.05	0.23 ± 0.09	0.22 ± 0.15		
*p*-value	<0.001	0.007	<0.001		
Hedges’ g (95% CI)	2.49 (1.62–3.51)	0.78 (0.22–1.38)	1.02 (0.68–1.43)		

Pre- and post-intervention data are demonstrated as mean ± StDev. * significant at *p* < 0.05, *η*^2^_Partial_: ANOVA’s effect size, Hedges**’** g: *t*-test’s effect size. ***Abbreviations****:* PLPT: paretic-limb-only plyometric training, DLPT: double-limb plyometric training, StepL: step length, SLSt: single-limb support-time, S-GSI: spatial gait symmetry-index, T-GSI: temporal gait symmetry-index.

## Data Availability

The data that support the findings of this study are available on reasonable request from the corresponding author.
